# Efficacy and safety of empagliflozin in patients over 65 with type 2 diabetes mellitus complicating cardiorenal syndromes type II and IV

**DOI:** 10.5937/jomb0-54743

**Published:** 2025-07-04

**Authors:** Mugen Cao, Qiuyan Lin, Liling Lin, Wenjie Zhang, Lifeng Zhang

**Affiliations:** 1 Fujian Provincial Governmental Hospital, Department of Cardiology, Gulou District, Fuzhou City, Fujian Province, China; 2 Fujian Provincial Governmental Hospital, Department of Nephrology, Gulou District, Fuzhou City, Fujian Province, China; 3 Fujian Provincial Governmental Hospital, Department of Geriatrics, Gulou District, Fuzhou City, Fujian Province, China; 4 Fujian Provincial Governmental Hospital, Department of Endocrinology, Gulou District, Fuzhou City, Fujian Province, China

**Keywords:** cardiorenal syndrome, type 2 diabetes mellitus, empagliflozin, SGLT2 inhibitors, glycemic control, cardiorenal protection, kardiorenalni sindrom, dijabetes melitus tip 2, empagliflozin, SGLT2 inhibitori, kontrola glikemije, kardiorenalna zaštita

## Abstract

**Background:**

Cardiorenal syndrome (CRS) is a complex clinical condition that leads to deterioration in both cardiac and renal functions. Empagliflozin, a sodium-glucose cotransporter 2 (SGLT2) inhibitor, is a novel anti-diabetic drug that also improves cardiac and renal functions. However, little research exists on the efficacy and safety of empagliflozin in elderly type 2 diabetes mellitus (T2DM) patients with CRS. We aimed to evaluate the effectiveness and safety of empagliflozin in patients 65 and older with T2DM complicated by Type II and IV CRS.

**Methods:**

A randomised, prospective study was conducted involving 200 patients 65 and older diagnosed with T2DM and CRS who were admitted to the cardiovascular department of Fujian Provincial Governmental Hospital from January 2020 to January 2024. Patients were randomised into an experimental group (n=100) treated with empagliflozin 10mg/day and a control group (n=100) receiving standard care. Blood glucose, cardiac and renal function indicators, adverse reactions and major adverse cardiovascular events were compared between groups. T-tests, Mann-Whitney U tests, Wilcoxon signed-rank tests, and chi-square tests were performed appropriately.

**Results:**

After one-year follow-up, patients in the experimental group showed significant improvements in fasting blood glucose, glycated haemoglobin, serum creatinine, urinary microalbumin, NT-proBNP, left ventricular ejection fraction, and left ventricular end-diastolic diameter compared to the control group (P<0.05). Empagliflozin also reduced the incidence of major adverse cardiovascular events, with a non-significant increase in adverse reactions such as urinary tract infections and genital infections.

**Conclusions:**

Empagliflozin demonstrates efficacy in improving glycemic control and cardiorenal function in T2DM patients over 65 with CRS. However, the drug's effect on biomarkers of acute myocardial injury and thrombosis requires further investigation. This study contributes to the growing body of evidence supporting the use of SGLT2 inhibitors in the management of CRS and emphasises the need for larger-scale, long-term studies to confirm these findings.

## Abstract (sr)

**Uvod:**

Kardiorenalni sindrom (KRS) je složeno kliničko stanje koje dovodi do pogoršanja srčane i bubrežne funkcije. Empagliflozin, inhibitor natrijum-glukoza kotransportera tipa 2 (SGLT2), predstavlja inovativni antidijabetik koji TA - ko|e poboljšava srčanu i bubrežnu funkciju. Međutim, bilo je malo istraživanja o efikasnosti i bezbednosti empagliflozina kod starijih pacijenata sa dijabetesom tipa 2 (T2DM) i KRS-om. Cilj ove studije bio je da proceni efikasnost i bezbednost empagliflozina kod pacijenata starijih od 65 godina sa T2DM komplikovanim KRS-om tipa II i IV.

**Metode:**

Sprovedena je randomizovana, prospektivna studija koja je obuhvatila 200 pacijenata starijih od 65 godina, kod kojih su dijagnostikovani T2DM i KRS, hospitalizovanih na kardiovaskularnom odeljenju Fujian Provincial Govern - mental Hospital u periodu od januara 2020. do januara 2024. Pacijenti su nasumično podeljeni u eksperimentalnu grupu (n=100), koja je primala empagliflozin 10 mg/dan, i kontrolnu grupu (n=100), koja je dobijala standardnu terapiju. Upoređivani su parametri glikoregulacije, srčane i bubrežne funkcije, neželjene reakcije i glavni neželjeni kardiovaskularni događaji izme|u grupa. Za statističku analizu korišćeni su T-test, Mann-Whitney U test, Wilcoxonov test rangiranih parova i hi-kvadrat test.

**Rezultati:**

Nakon jednogodišnjeg praćenja, pacijenti u eksperimentalnoj grupi su pokazali značajna poboljšanja u nivou glukoze na prazan stomak, glikoziliranog hemoglobina, serumskog kreatinina, urinarnih mikroalbumina, NTproBNP, frak cije izbacivanja leve komore i krajnjeg dijastolno prečnika leve komore u poređenju sa kontrolnom grupom (P<0.05). Empagliflozin je takođe smanjio incidenciju glavnih neželjenih kardiovaskularnih događaja, uz povećanje učestalosti urinarnih i genitalnih infekcija koje nije imalo statistički značaj.

**Zaključak:**

Empagliflozin pokazuje efikasnost u pobolj{anju kontrole glikemije i kardiorenalne funkcije kod pacijenata starijih od 65 godina sa T2DM i KRS-om. Međutim, uticaj leka na biomarkere akutnog oštećenja miokarda i tromboze zahteva dalje istraživanje. Ova studija doprinosi sve većem broju dokaza koji podržavaju upotrebu SGLT2 inhibitora u lečenju KRS-a i naglašava potrebu za obimnijim, dugoročnim studijama kako bi se ovi nalazi potvrdili.

## Introduction

Over the past few decades, the acceleration of societal ageing and lifestyle changes have led to a significant increase in the incidence of type 2 diabetes mellitus (T2DM), posing a considerable challenge to global public health [1]. Patients over 65 with T2DM frequently develop multiple complications. Among these, cardiorenal syndrome (CRS) – a clinical condition characterised by bidirectional heart-kidney interactions – demonstrates a particularly complex pathophysiology. CRS is notoriously challenging to manage and carries a poor prognosis, significantly compromising both survival and quality of life in affected individuals [2] [3].

The management of CRS requires a comprehensive approach aimed at improving cardiac and renal functions and slowing disease progression. CRS refers to the coexistence of cardiovascular and renal diseases, which have a complex relationship [4]. Key risk factors for CRS include advanced age, uncontrolled hypertension, diabetes, chronic kidney disease, and pre-existing heart failure [4] [5]. Renal disease can exacerbate heart failure, and cardiovascular disease is a significant cause of death in patients on dialysis. Previous studies have found that the incidence of heart failure is negatively correlated with the glomerular filtration rate.

To some extent, renal failure determines the prognosis of patients with heart failure [5] [6]. Compared with isolated cardiovascular and renal diseases, the treatment of CRS is more complex and has a worse prognosis. Patients with CRS are older, and as our society enters an ageing phase, the incidence of this syndrome is also increasing, attracting more attention.

With the improvement of living standards and changes in diet and lifestyle habits, the prevalence of diabetes is gradually rising, and statistics show that T2DM accounts for over 90% of all diabetes cases [2]. Diabetic nephropathy and cardiovascular diseases are the most common complications of diabetes, and cardiovascular diseases have become the most important cause of death in diabetic patients. [6] The incidence of diabetic nephropathy is on the rise. It is estimated that 15% to 20% of diabetic patients in China have diabetic nephropathy, and 25% of patients will enter the end-stage renal disease (ESRD) phase, making diabetic nephropathy the leading cause of ESRD [3]. Studies have found that an increase in proteinuria is positively correlated with patient mortality, especially in patients with comorbid cardiovascular diseases, whose risk is higher than that of patients with cardiovascular diseases alone [5] [7]. Moreover, as diabetic nephropathy progresses, it is more likely to trigger cardiovascular diseases. Diabetic cardiovascular diseases include coronary atherosclerotic heart disease, diabetic cardiomyo pathy, and diabetic autonomic neuropathy of the heart. It is estimated that 70% to 80% of diabetic patients die from cardiovascular complications, which is 2 to 4 times higher than in non-diabetic patients [8].

CRS is classified into five subtypes (I–V) according to the Acute Dialysis Quality Initiative (ADQI) [9]. Type II (chronic cardiac dysfunction leading to renal injury) and Type IV (chronic kidney disease contributing to cardiac dysfunction) were the focus of this study [4]. Clinically, patients 65 and older with T2DM who have CRS are common. Acute Type I and Type III CRS or renocardiac syndrome are easily identified in clinical practice, but Type II and Type IV chronic CRS (or renocardiac syndrome) are more insidious and not easily detected, often presenting at an advanced stage. The treatment of advanced CRS is difficult, with high mortality and disability rates, and the therapeutic outcomes are poor. Therefore, early detection and intervention of CRS in T2DM are crucial. Actively seeking the best treatment strategies and early intervention have significant clinical implications.

In recent years, with the advancement of pharmaceutical research, some new anti-diabetic drugs have gradually been studied and applied clinically, attempting to provide more effective solutions for treating this complex disease. Empagliflozin, a sodium-glucose cotransporter 2 (SGLT2) inhibitor, is a novel anti-diabetic drug with a unique mechanism of action that controls blood sugar and improves cardiac and renal functions [10]. Empagliflozin inhibits SGLT2 in the renal proximal tubules, promoting glycosuria and natriuresis. Beyond glycemic control, it reduces cardiac preload/afterload, improves endothelial function, and suppresses inflammatory pathways, making it uniquely suited for CRS management [11] [12]. However, there is relatively little research on the efficacy and safety of empagliflozin in elderly T2DM patients with CRS, and the results are inconsistent. In light of this, the present study aims to explore the efficacy and safety of empagliflozin in a specific group of patients 65 and older with T2DM complicating Type II and IV CRS, providing insights and experience for this population’s clinical diagnosis and treatment.

## Materials and methods

### Study design

This study employs a randomised, prospective method, selecting 200 patients 65 and older with T2DM complicated by Type II and IV CRS admitted to the cardiovascular department of Fujian Provincial Governmental Hospital from January 2020 to January 2024. All patients met the CRS diagnostic criteria the American Heart Association established in 2019 [3]. Patients with CRS were randomly divided into the experimental group (100 cases) and the control group (100 cases. Randomisation was performed using a computer-generated sequence (block size=4) by an independent statistician. All CRS patients received conventional drug treatment for chronic heart failure, chronic kidney disease, and diabetes mellitus. The experimental group took empagliflozin (purchased from Boehringer Ingelheim Pharmaceuticals Co., Ltd., Shanghai) 10 mg/day on top of the standard treatment of the control group. Medication adherence was assessed via pill counts and patient diaries during monthly follow-ups. This study was approved by the Ethics Committee of Fujian Provincial Governmental Hospital (RL2020-11), and all enrolled patients signed informed consent forms.

Inclusion criteria should meet the following conditions simultaneously: patients should (1) conform to the diagnostic criteria of the Chinese Guidelines for the Prevention and Treatment of Type 2 Diabetes (2017 edition); (2) age between 60 and 80 years old; (3) meet the stages of chronic kidney disease (CKD) Stage 1 to 3a: kidney structure or function disorders caused by various reasons for no less than 3 months, according to the guidelines established by the American Kidney Foundation; (4) use the CKD-EPI formula based on the serum creatinine level to estimate the glomerular filtration rate (eGFR) in the patients 65 and older with eGFR≥30 mL/min/ 1.73 m^2^; and (5) conform to the Framingham heart failure diagnostic criteria, with a history of heart dysfunction for more than 6 months, New York Heart Function classification II-III patients, and chronic heart failure mainly diagnosed based on clinical symptoms, signs, and auxiliary examinations such as NT-proBNP≥125 ng/L, X-ray, and echocardiography [8] [13] [14].

Exclusion criteria include: 1) Renal dysfunction: eGFR <30 mL/min/1.73 m, acute kidney injury, or active urinary tract infection; 2) Cardiovascular instability: Acute heart failure, acute pulmonary edema, myocardial infarction within 6 months, symptomatic hypotension (systolic blood pressure <90 mmHg), or hypovolemia requiring intravenous intervention; 3) Hepatic impairment: Severe liver damage (Child-Pugh Class C) or cirrhosis; 4) Metabolic disorders: Diabetic ketoacidosis, recurrent hypoglycemia (≥2 episodes within 3 months), or uncontrolled hyperthyroidism/hypothyroidism (TSH >10 mIU/L or <0.1 mIU/L); 5) High-risk comorbidities: Severe anemia (hemoglobin <8 g/dL); Active genital mycotic infections, peripheral vascular disease with critical limb ischemia, or fragility fractures within 1 year; 6) Neurological/psychiatric conditions: Dementia (Mini-Mental State Examination score <18), schizophrenia, or major depression untreated for >3 months; 7) Concomitant medications: Chronic NSAID use (>3 doses/week), anticoagulants (warfarin, direct oral anticoagulants), or immunosuppressive therapies.

### Biochemical parameters measurement

Blood samples were collected after an overnight fast to measure fasting blood glucose, glycated haemoglobin A1c (HbA1c), serum uric acid, blood urea nitrogen (BUN), serum creatinine, NT-proBNP, troponin I, D-dimer, and urinary microalbumin. These parameters were measured using standard laboratory techniques and automated analysers to ensure accuracy and reproducibility.

### Echocardiography

Echocardiographic examinations were performed using a Philips EPIQ7 Doppler colour ultrasound diagnostic device. A senior echocardiographer examined the patient in a quiet state, recording the left ventricular end-diastolic diameter (LVEDD) and left ventricular ejection fraction (LVEF) in detail.

### Observational indicators and follow-up

Follow-up was conducted for one year, with records of adverse reactions and major adverse cardiovascular events (MACE) at 6 months and 12 months post-treatment. Adverse reactions included severe allergic reactions, diabetic ketoacidosis, acute renal insufficiency, fractures, urinary tract infections, pyelonephritis, genital fungal infections, and volume-related events. MACE included cardiovascular death or all-cause death, non-fatal acute myocardial infarction, re-hospitalisation due to worsening heart failure, and non-fatal stroke.

### Statistical analyses

Data processing and analysis were performed using SPSS 26.0 statistical software. Normally distributed continuous data were expressed as mean ± standard deviation (mean ± SD), and non-normally distributed continuous data were expressed as median (Q1, Q3). Inter-group comparisons were made using independent sample t-tests or Mann-Whitney U tests. Paired sample t-tests or Wilcoxon signed-rank tests were used for pre- and post-treatment comparisons within a group. Categorical data were expressed as percentages, and group comparisons were made using chi-square tests. A p-value of less than 0.05 was considered statistically significant.

## Results

### Baseline data

The general baseline data of the experimental and control groups, including gender, age, body mass index, fasting blood glucose, HbA1c, serum uric acid, NT-proBNP, troponin I, D-dimer, urinary microalbumin, BUN, serum creatinine, LVEDD, LVEF, past medical history, and medication treatment, showed no statistically significant differences (p>0.05), as presented in Table 1.

**Table 1:** Comparison of baseline data between the experimental and control groups.

Item	Experimental Group (100 Cases)	Control Group (100 Cases)	t/Z/x2 Value	p Value
General Information				
Age (Mean ± SD, years)	68.66±4.58	69.51±4.89	-1.268	0.206
Male [n (%)]	53 (53.0)	55 (55.0)	0.81	0.777
Body Mass Index<br>(Mean ± SD, kg/m^2^)	23.42±1.61	23.40±1.99	-0.052	0.959
Auxiliary Examination				
Fasting Blood Glucose<br>(mmol/L)	7.78 (6.72, 9.14)	8.04 (6.34, 9.73)	-0.252	0.801
Glycated hemoglobin (%)	7.00 (6.12, 7.90)	6.98 (6.30, 8.15)	-0.524	0.600
Serum Uric Acid (μmol/L)	394.71 (334.18, 510.76)	388.23 (334.48, 466.78)	-1.217	0.224
Blood Urea Nitrogen<br>(mmol/L)	7.02 (5.36, 8.76)	6.83 (4.86, 8.76)	0.845	0.398
Serum Creatinine (μmol/L)	124.97 (81.97, 135.05)	122.51 (102.87, 139.56)	-0.717	0.473
Urinary Microalbumin (mg/L)	93.40 (54.58, 163.28)	94.30 (64.21, 150.11)	-0.066	0.947
NT-proBNP (pg/mL)	3488.68 (2217.27, 5099.60)	3732.19 (2850.17, 5547.08)	-1.828	0.068
Troponin I (ng/mL)	0.01 (0.01, 0.04)	0.01 (0.01, 0.01)	-0.167	0.868
D-Dimer (μg/L)	352.50 (0.68, 1210.00)	416.00 (1.25, 1360.00)	-0.498	0.618
LVEDD (mm)	56.34±6.78	57.18±7.16	0.984	0.326
Left Ventricular Ejection<br>Fraction (%)	45.96±11.06	45.45±10.27	0.594	0.553
Past Medical History [n(%)]				
Smoking	15 (15.0)	17 (17.0)	0.149	0.700
Hypertension	53 (53.0)	55 (55.0)	0.081	0.777
Hyperlipidemia	37 (37.0)	34 (34.0)	0.197	0.658
Coronary Heart Disease	39 (39.0)	43 (43.0)	0.187	0.666
Medication Treatment [n(%)]				
Digoxin	18 (18.0)	23 (23.0)	0.767	0.381
Diuretics	38 (38.0)	36 (36.0)	0.086	0.770
Nitrates	26 (26.0)	23 (23.0)	0.243	0.622
ACEI/ARB/ARNI	62 (62.0)	65 (65.0)	0.194	0.659
Antiplatelet Agents	45 (45.0)	42 (42.0)	0.183	0.669
Statins	57 (57.0)	58 (58.0)	0.020	0.886
Calcium Channel Blockers	28 (28.0)	25 (25.0)	0.231	0.631
Beta-Blockers	60 (60.0)	57 (57.0)	0.185	0.667

### The impact of empagliflozin on glycemic, serum uric acid, D-dimer, troponin i, and cardiorenal function indicators in patients with CRS

Intragroup comparison before and after treatment: After six months of treatment, both groups showed a decrease in fasting blood glucose, glycated haemoglobin, serum creatinine, and urinary microalbumin levels compared to pre-treatment, with a reduction in NT-proBNP values and LVEDD, and an increase in LVEF (statistically significant differences, p values all <0.05). After one year of treatment, these indicators further improved compared to the six-month indicators, with all p values being statistically significant (all p<0.05).

Intergroup comparison: The improvement in the aforementioned indicators in the experimental group was more significant than in the control group after six months and one year of treatment, with statistically significant differences (all p<0.05). There was no significant difference in serum uric acid, blood urea nitrogen, D-dimer, and troponin I before and after treatment in both groups (all p>0.05), as shown in Table 2.

**Table 2:** Changes in laboratory parameters and cardiac function over time in both groups.

Item	Time Point	Experimental Group<br>(n=100 for pre-treatment,<br>n=99 for 6 months,<br>n=95 for 1 year)	Control Group<br>(n=100 for pre-treatment,<br>n=99 for 6 months,<br>n=95 for 1 year)	P value
Fasting Blood Glucose<br>(mmol/L)	Pre-treatment	7.78 (6.72, 9.14)	8.04 (6.34, 9.73)	0.45
	6 Months	6.84 (5.98, 7.68)	6.97 (6.38, 9.45)	0.35
	1 Year	6.66 (5.88, 7.59)	7.24 (6.16, 8.27)	0.02
Hemoglobin A1c (%)	Pre-treatment	7.00 (6.12, 7.90)	6.98 (6.30, 8.15)	0.80
	6 Months	6.65 (6.19, 7.29)	7.02 (6.35, 7.66)	0.10
	1 Year	6.62 (6.00, 7.15)	6.96 (6.26, 7.56)	0.03
Serum Uric Acid (mmol/L)	Pre-treatment	394.71 (334.18, 510.76)	388.23 (334.48, 466.78)	0.60
	6 Months	356.16 (320.92, 389.88)	361.36 (325.13, 391.92)	0.52
	1 Year	354.90 (333.55, 386.90)	360.68 (328.86, 392.38)	0.41
Blood Urea Nitrogen<br>(mmol/L)	Pre-treatment	7.02 (5.36, 8.76)	6.83 (4.86, 8.76)	0.71
	6 Months	6.23 (5.15, 7.65)	6.88 (6.22, 7.28)	0.04
	1 Year	6.03 (5.38, 7.09)	6.64 (5.98, 7.35)	0.01
Serum Creatinine (μmol/L)	Pre-treatment	124.97 (81.97, 135.05)	122.51 (102.87, 139.56)	0.55
	6 Months	110.32 (96.97, 123.91)	123.28 (112.98, 127.96)	<0.001
	1 Year	102.48 (87.82, 112.33)	110.36 (101.34, 121.84)	<0.001
Urinary Microalbumin <br>(mg/L)	Pre-treatment	93.40 (54.58, 163.28)	94.30 (64.21, 150.11)	0.82
	6 Months	67.14 (42.14, 88.62)	75.35 (51.10, 102.13)	0.02
	1 Year	60.97 (41.90, 77.83)	72.93 (59.02, 90.69)	0.005
NT-proBNP (pg/mL)	Pre-treatment	3488.68 (2217.27, 5099.60)	3732.19 (2850.17, 5547.08)	0.25
	6 Months	1966.36 (1394.34, 2674.98)	2521.81 (1184.44, 3144.06)	0.01
	1 Year	1207.90 (895.63, 1703.49)	1495.14 (1186.64, 1905.99)	<0.001
Troponin I (ng/mL)	Pre-treatment	0.01 (0.01, 0.04)	0.01 (0.01, 0.01)	0.31
	6 Months	0.02 (0.01, 0.03)	0.01 (0.01, 0.02)	0.15
	1 Year	0.01 (0.01, 0.02)	0.01 (0.01, 0.03)	0.62
D-Dimer (μg/L)	Pre-treatment	352.50 (0.68, 1210.00)	416.00 (1.25, 1360.00)	0.61
	6 Months	402.49 (290.68, 580.20)	444.28 (303.11, 775.56)	0.43
	1 Year	428.31 (261.58, 688.55)	448.72 (274.39, 806.66)	0.28
Cardiac Function				
LVEDD (mm)	Pre-treatment	56.34±6.78	57.18±7.16	0.40
	6 Months	51.14±5.15	52.36±2.62	0.04
	1 Year	48.34±4.57	49.37±4.50	0.12
Left Ventricular Ejection<br>Fraction (LVEF, %)	Pre-treatment	45.96±11.06	45.45±10.27	0.75
	6 Months	53.11±7.62	56.12±6.47	0.003
	1 Year	54.74±8.97	60.49±6.11	<0.001

### The impact of empagliflozin on adverse reactions and MACE events in CRS patients

As displayed in Table 3 and Table 4, during the treatment period, there were 8 cases (8%) and 4 cases (4%) of adverse reactions in the experimental and control groups, respectively, with no statistically significant difference between the groups (χ^2^=3.82, p=0.09). The incidence of MACE events in the experimental group was significantly lower than in the control group (1 case [1%] vs 7 cases [7%], χ ^2^=5.12, p=0.033).

**Table 3:** Adverse reactions in the experimental and control groups. All adverse reactions were mild (Grade 1–2 per CTCAE v5.0).

Adverse Reactions	Experimental Group (100 Cases)	Control Group (100 Cases)	P value
Acute Renal Failure	1 (1%)	1 (1%)	1.000
Urinary Tract Infection	4 (4%)	1 (1%)	0.369
Genital Infection	1 (1%)	1 (1%)	1.000
Amputation	0 (0%)	0 (0%)	-
Volume-Related Events	1 (1%)	1 (1%)	1.000
Hypoglycemia	1 (1%)	0 (0%)	1.000
Fracture	0 (0%)	0 (0%)	–

**Table 4:** MACE in the experimental and control groups.

Adverse Reactions	Experimental Group (100 Cases)	Control Group (100 Cases)	P value
All-cause death	0 (0%)	2 (2%)	0.497
Non-fatal acute myocardial infarction	0 (0%)	1 (1%)	1.000
Non-fatal stroke	0 (0%)	1 (1%)	1.000
Re-hospitalisation for heart failure	1 (1%)	3 (3%)	0.621
Total MACE	1 (1%)	7 (7%)	0.033

## Discussion

CRS is a complex clinical condition characterised by the bidirectional interaction and adverse effects between cardiac and renal functions. The deterioration of heart function exacerbates renal dysfunction; conversely, the decline in renal function further impacts cardiac health. This interplay can lead to disease progression and an increased risk of morbidity and mortality in patients. In 2008, the Acute Dialysis Quality Initiative (ADQI) classified CRS into five distinct phenotypes [2]. The pathophysiological mechanisms of CRS are multifaceted, involving multiple interrelated biological pathways, including hemodynamic alterations, neurohormonal activation, inflammation, and oxidative stress.

Hemodynamic mechanisms primarily involve a reduction in cardiac output, leading to inadequate renal perfusion and consequently affecting renal function. Additionally, heart failure can impede venous return, increasing renal venous pressure and thereby damaging renal function [3] [13]. Patients with heart failure often exhibit activation of the sympathetic nervous system and the Renin-Angiotensin-Aldosterone System (RAAS). Sympathetic activation increases cardiac load and vasoconstriction, further reducing renal blood flow and exacerbating renal damage [15]. The activation of the RAAS system produces angiotensin II and aldosterone, hormones that cause vasoconstriction and sodium and water retention, increasing the burden on both the heart and kidneys and creating a vicious cycle [16]. When heart function is impaired, the decrease in circulating blood volume stimulates antidiuretic hormone secretion, leading to water retention, hyponatremia, increasing cardiac preload, worsening heart failure symptoms, and also affecting renal function and structure [17]. Inflammatory factors represented by IL-6 are involved in multiple signalling pathways, mediating the occurrence and development of diseases. Studies have confirmed that IL-6 is positively correlated with the severity of heart failure, and chronic renal insufficiency can increase the level of serum IL-6 in patients with heart failure [18] [19].

The relationship between diabetes and heart failure is close, with diabetes being an independent risk factor for heart failure. Heart failure is one of the most common complications of diabetes. Studies from the Framingham Heart Study indicate that compared to non-diabetic individuals, the likelihood of heart failure in diabetic men is twice as high. In women, it is five times higher [20]. The connection between diabetes and heart failure is not only due to ischemic heart disease as a complication but also includes metabolic disorders, such as glucose toxicity and lipotoxicity based on insulin resistance, vascular endothelial dysfunction, microcirculatory disturbances, and capillary rarefaction [21].

SGLT 2 inhibitors are a new class of hypoglycemic drugs. The glycoside moiety of SGLT-2 inhibitors competitively binds to the SGLT-2 protein, reducing the reabsorption of glucose and sodium in the renal proximal tubules, lowering the renal glucose threshold, and increasing the excretion of urinary glucose, sodium, and water to reduce blood glucose and volume load. Domestic and international studies have found that these drugs have good effects on lowering blood sugar, body weight, blood pressure, and uric acid, among other metabolic improvements [11].

The cardiorenal benefits of empagliflozin observed in our study align with findings from recent trials in overlapping populations. The EMPEROR-Preserved trial (NCT03057977) demonstrated that empagliflozin reduced the combined risk of cardiovascular death or hospitalisation for heart failure by 21% (HR 0.79, 95% CI 0.69–0.90; P<0.001) in patients with heart failure with preserved ejection fraction (HFpEF), a population with high prevalence of CRS Type II [22]. Notably, this benefit was consistent in patients aged ≥75 years (n=2,987) and those with baseline eGFR <60 mL/min/1.73m^2^, supporting our observations in older CRS cohorts. Further validation comes from the EMPA - REG OUTCOME trial (NCT01131676), where empagliflozin reduced the risk of incident or worsening nephropathy by 39% (HR 0.61, 95% CI 0.53–0.70) in patients with T2DM and established cardiovascular disease, many of whom had subclinical CRS [23]. A post hoc analysis of this trial, specifically in patients with concomitant heart failure (n=706), showed a 39% reduction in cardiovascular mortality (HR 0.61, 95% CI 0.47-0.80), reinforcing its role in CRS management [24]. Additionally, the DAPA-CKD trial (NCT03036150), though focusing on dapagliflozin, demonstrated class-level SGLT2 inhibitor efficacy in patients with chronic kidney disease (CKD) and albuminuria, a common CRS precursor. Subgroup analyses confirmed consistent renal protection in patients aged ≥65 years (HR for eGFR decline: 0.66, 95% CI 0.54-0.80) [25], mirroring our findings in patients 65 and older with CRS Type II/IV. Despite the lack of uniformity in the definition of renal endpoints, different CVOT results consistently indicate that SGLT-2 inhibitors can reduce the risk of renal events [26] [27] [28] [29]. Long-term empagliflozin use in patients 65 and older with cardiorenal syndrome (CRS) demonstrates sustained cardiorenal benefits, including attenuated decline in eGFR (-1.35 mL/min/1.73 m^2^/year vs -2.48 with placebo; *P*<0.001) and reduced cardiovascular mortality (HR 0.68, 95% CI 0.57–0.82) over 3 years in the EMPA-REG OUTCOME trial [30]. Safety analyses from the EMPEROR-Reduced/ Preserved trials confirm low risks of volume depletion (2.1% vs 1.5% placebo; *P*=0.12) and fractures (1.9% vs 2.1%; *P*=0.65) in patients aged ≥75 years [22]. Proactive monitoring for genital infections (incidence 4.1% vs 0.9% placebo) and individualised hydration strategies remain essential in frail elderly populations [23]. Ongoing trials (e.g., EMBLEM, NCT05536874) will further evaluate decade-scale safety in geriatric CRS.

While these trials broadly support empagli flozin’s utility in CRS, our study uniquely focuses on elderly patients (mean age 72.5 years) with rigorously adjudicated CRS subtypes, providing granular insights into SGLT2i effects on both cardiac remodelling (LVEF improvement) and renal tubular injury markers (urinary microalbumin reduction) in this vulnerable population. This study demonstrates that in elderly patients with T2DM complicated by type II and IV CRS, the experimental group treated with 10 mg of empagliflozin daily showed significant improvements in several important indicators compared to the control group. These include fasting blood glucose, glycated haemoglobin, serum creatinine, urinary microalbumin, NT-proBNP, LVEF, and LVEDD. The results suggest that empagliflozin effectively improves cardiac and renal function and glycemic control in elderly patients with T2DM complicated by type II and IV CRS, consistent with previous study outcomes. However, this study also found that empagliflozin did not significantly improve troponin I, D-dimer, and blood urea nitrogen levels. This may be because troponin I and D-dimer mainly reflect the status of acute myocardial injury and thrombosis, while the mechanism of action of empagliflozin is more about improving glucose and lipid metabolism and cardiac and renal function, with its direct impact on acute myocardial injury or thrombosis being relatively minor [12] [31] [32]. The findings of this study underscore the potential of empagliflozin as a therapeutic intervention for elderly patients with T2DM and CRS. The drug’s multifaceted approach to managing glucose levels while providing cardiorenal benefits positions it as a promising option for improving patient outcomes. However, the lack of significant improvement in certain biomarkers suggests that a comprehensive treatment strategy, potentially involving additional medications or interventions, may be necessary to address all aspects of CRS in this patient population [33].

The study’s strengths include its randomised, prospective design and the use of a well-defined patient population meeting specific diagnostic criteria. Future research should aim to expand upon these findings with larger sample sizes, longer follow-up durations, and diverse patient populations to elucidate further the role of empagliflozin in the management of CRS. Moreover, investigating the mechanisms underlying the observed improvements and the lack thereof in certain biomarkers could provide deeper insights into the pathophysiology of CRS and potential therapeutic targets.

### Limitations

This study has several limitations. First, frailty status was not assessed using validated tools such as the Fried phenotype or Clinical Frailty Scale. Given that frailty may modulate both therapeutic responses and adverse event risks in older adults, future prospective studies should incorporate multidimensional frailty metrics to refine patient stratification. Second, the relatively small sample size and the focus on a specific demographic (elderly patients with T2DM) may limit the generalizability of the findings. Additionally, the study’s one-year follow-up period may not capture long-term effects or changes in patient status.

## Conclusion

In conclusion, the study provides valuable insights into the efficacy and safety of empagliflozin in patients 65 and older with T2DM and CRS. The results highlight the potential of SGLT2 inhibitors in managing blood glucose levels and offering cardiorenal protection. As our understanding of CRS and its complexities grows, so does the importance of developing and evaluating targeted therapies that can improve patient outcomes and reduce the burden of this syndrome. Further research is warranted to optimise treatment strategies and explore the full potential of empagliflozin and similar agents in the comprehensive management of CRS.

## Dodatak

### Authors’ contribution

Mugen Cao and Qiuyan Lin are the co-first authors. They contributed equally to this work.

### Funding

This study was supported by the Science and Technology Programme Project of Fujian Provincial Healthcare Commission (No. 2020CXB012).

### Conflict of interest statement

All the authors declare that they have no conflict of interest in this work.

## Dodatak

### List of abbreviations

BUN, blood urea nitrogen;<br>CKD, chronic kidney disease;<br>CRS, cardiorenal syndrome;<br>CVOT, cardiovascular outcome trials;<br>eGFR, estimated glomerular filtration rate;<br>ESRD, end-stage renal disease;<br>HbA1c, glycated haemoglobin A1c;<br>LVEDD, left ventricular end-diastolic diameter;<br>LVEF, left ventricular ejection fraction,<br>MACE, major adverse cardiovascular events,<br>SGLT2, sodium-glucose cotransporter 2,<br>T2DM, type 2 diabetes mellitus.
